# Dual-channel endoscope for double-traction endoscopic device-assisted full-thickness resection of rectal superficial tumor

**DOI:** 10.1055/a-2316-3626

**Published:** 2024-05-17

**Authors:** Giuseppe DellʼAnna, Francesco Vito Mandarino, Paolo Biamonte, Francesca Bernardi, Vito Annese, Silvio Danese, Francesco Azzolini

**Affiliations:** 1 Gastroenterology and Gastrointestinal Endoscopy Unit, IRCCS San Raffaele Institute, Milan, Italy; 2 27288 Gastroenterology and Gastrointestinal Endoscopy Unit, IRCCS Policlinico San Donato, Milan, Italy; 3 Vita-Salute San Raffaele University, Milan, Italy

Endoscopic device-assisted full-thickness resection (EDFTR) with over-the-scope clip deployment is a novel technique for treating complex colorectal polyps, specifically nonlifting adenomas (recurrent or previously biopsied/tattooed) or early carcinomas 1 . For these lesions, EDFTR has demonstrated a high technical success rate, and a good efficacy and safety profile 2 3 . The technical success of EDFTR may be hindered by lesions with significant fibrosis that cannot be adequately lifted even when using dedicated grasping forceps 1 4 .

We recently managed a case involving a 74-year-old woman who was diagnosed, during a screening colonoscopy in another hospital, with a 15-mm-diameter rectal nongranular laterally spreading tumor (LST-NG). The lesion was extensively biopsied., Evaluation by digital chromoendoscopy (I-SCAN; Pentax Medical, Tokyo, Japan) revealed that the LST-NG had a pseudodepressed central area (0-IIa+0-IIc according to the Paris Classification) with pit pattern IV, according to the Kudo Classification ( Fig. 1 ). After a multidisciplinary discussion of all alternatives, EDFTR was proposed 5 ( Video 1 ).

**Fig. 1:**
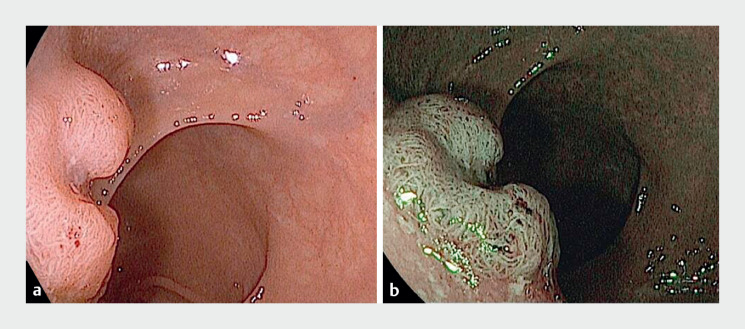
Endoscopic evaluation of the rectal lesion revealed a 15-mm rectal nongranular laterally spreading tumor (LST-NG) with a pseudodepressed central area (0-IIa + 0-IIc according to the Paris Classification), characterized by pit pattern IV according to the Kudo Classification. **a** White-light endoscopy. **b** Virtual chromoendoscopy with I-SCAN technology (Pentax Medical, Tokyo, Japan).

Double-traction endoscopic device-assisted full thickness resection.Video 1

Owing to the presence of severe fibrosis, adequate traction of the lesion could not be achieved either with suction or with a full-thickness resection device (FTRD; Ovesco Endoscopy, Tübingen, Germany) grasping forceps.

Subsequently, the FTRD was mounted onto a dual-channel (3.7 mm and 2.8 mm in size) therapeutic gastroscope (GIF-2TH180; Olympus, Tokyo, Japan) ( Fig. 2 ). First, the lesion was marked using a dedicated probe. To aid traction, two foreign body forceps (one for each operating channel) were simultaneously used to gently pull the lesion into the FTRD distal cap. Subsequently, an over-the-scope clip was released, and the lesion was resected “en bloc” by the FTRD diathermic snare. Finally, no residual tissue was seen on the resection base ( Fig. 3 ). No complications were recorded. The final histology showed a tubular adenoma with high grade dysplasia (R0 resection).

**Fig. 2:**
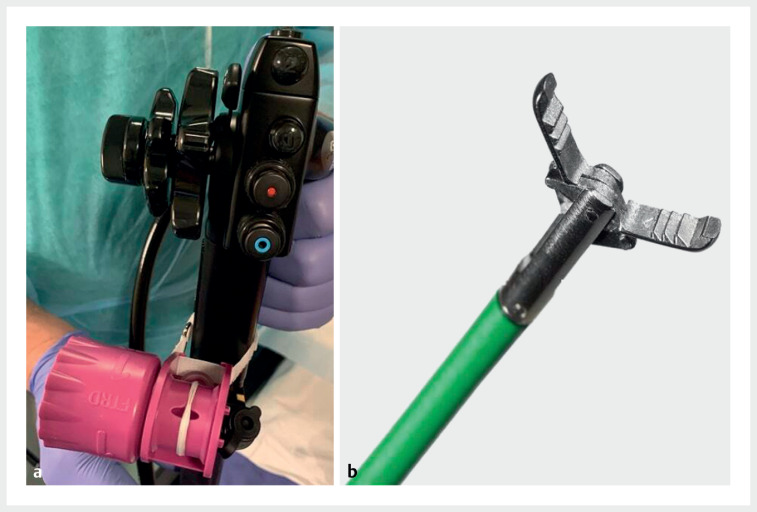
Dual-channel therapeutic gastroscope for double-traction endoscopic device-assisted full-thickness resection. The severe fibrosis resulting from previous biopsies prevented complete traction of the lesion using standard methods. **a** To achieve complete traction of the lesion into the distal cap of the full-thickness resection device (Ovesco Endoscopy, Tübingen, Germany), a dual-channel therapeutic gastroscope was used (GIF-2TH180; Olympus, Tokyo, Japan). **b** The two operating channels were employed to use two foreign body forceps for lesion traction.

**Fig. 3:**
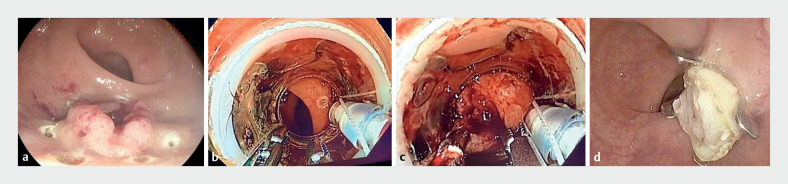
Double-traction endoscopic device-assisted full-thickness resection. **a** The lesion was marked using a dedicated marking probe. **b** Two foreign body forceps were used, one in each of the two operating channels of the endoscope. **c** The forceps were used simultaneously to pull the entire lesion into the distal cap of the full-thickness resection device. **d** Following the release of the over-the-scope clip, the lesion was resected en bloc with the diathermic snare.

In expert hands, double traction through a dual-channel endoscope could represent an additional tool for the treatment of challenging fibrotic polyps by EDFTR.

Endoscopy_UCTN_Code_CPL_1AJ_2AD_3AF

## Notes

Endoscopy E-Videos https://eref.thieme.de/e-videos
*E-Videos* is an open access online section of the journal *Endoscopy* , reporting on interesting cases and new techniques in gastroenterological endoscopy. All papers include a high-quality video and are published with a Creative Commons CC-BY license. Endoscopy E-Videos qualify for HINARI discounts and waivers and eligibility is automatically checked during the submission process. We grant 100% waivers to articles whose corresponding authors are based in Group A countries and 50% waivers to those who are based in Group B countries as classified by Research4Life (see: https://www.research4life.org/access/eligibility/ ). This section has its own submission website at https://mc.manuscriptcentral.com/e-videos .
